# Extended versus non-extended lymphadenectomy during radical cystectomy for patients with bladder cancer: a meta-analysis of the effect on long-term and short-term outcomes

**DOI:** 10.1186/s12957-019-1759-5

**Published:** 2019-12-21

**Authors:** Yu-Chen Wang, Jie Wu, Bo Dai, Yi-Jun Shen, Chun-Guang Ma, Ding-Wei Ye, Yi-Ping Zhu

**Affiliations:** 1 Department of Urology, Fudan University Shanghai Cancer Center, No. 270 Dong an Road, Shanghai, 200032 People’s Republic of China; 2 Department of Oncology, Shanghai Medical College, Fudan University, Shanghai, People’s Republic of China

## Abstract

**Background:**

Pelvic lymphadenectomy (PLND) is an integral part of curative surgery for high-risk non-muscle invasive and muscle-invasive bladder cancer. The therapeutic value of extended PLND is controversial.

**Methods:**

We conducted a comprehensive online search in PubMed, EMBASE, and the Cochrane Library databases for relevant literature directly comparing extended PLND (e-PLND) with non-extended PLND (ne-PLND) from database inception to June 2019. We performed the meta-analysis to evaluate the impact of PLND templates on recurrence-free survival (RFS), disease-specific survival (DSS), overall survival (OS), rates of postoperative major complications, and mortality within 90 days of surgery.

**Results:**

A total of 10 studies involving 3979 patients undergoing either e-PLND or ne-PLND were included. The results showed that e-PLND was significantly associated with better RFS (HR 0.74, 95% CI 0.62–0.90, *p* = 0.002) and DSS (HR 0.66, 95% CI 0.55–0.79, *p* < 0.001). However, no correlation was found between e-PLND template and a better OS (HR 0.93, 95% CI 0.55–1.58, *p* = 0.79). Postoperative major complications were similar between e-PLND group and ne-PLND group, as was mortality within 90 days of surgery.

**Conclusion:**

e-PLND template is correlated with favorable RFS and DSS outcomes for patients with bladder cancer. e-PLND did not have more postoperative major complications than did ne-PLND.

## Introduction

Lymph node dissection (LND) is an integral part of curative radical cystectomy (RC) for patients with muscle-invasive bladder cancer (MIBC). It is estimated that nearly one fourth of patients with MIBC possess lymph node metastases. Meticulous pelvic lymphadenectomy (PLND) increases staging procedure accuracy and possibly improves prognosis for both node-negative and node-positive patients [1]. However, results from studies focused on associations among LND templates and prognosis have been inconsistent, and hence, the therapeutic value of LND is debatable.

Several parameters, including the number of lymph nodes dissected, lymph node density, and LND template have been assessed to define adequate LND. Large amounts of studies have demonstrated the prognostic value of lymph node number removed by lymphadenectomy during RC. However, contradictory findings and substantial heterogeneity among these studies have questioned the usefulness of lymph node number as a measure to assess the adequacy of LND [2–4]. Therefore, a standardized LND template during RC would not only improve the quality of curative RC, but would also have a positive effect on outcomes for selected patients.

The major rationale for simultaneous PLND during RC is elimination of lymph node micro-metastases of MIBC cannot be clinically detected. Previous mapping studies revealed that half of patients with lymph node metastases also had positive nodes above the level of the iliac bifurcation [4–6]. For this group of patients, whether extended PLND (e-PLND) above the common iliac provides survival benefits in addition to adjuvant therapy or not is debatable.

Currently, there are two meta-analyses that support e-PLND is superior to non-extended PLND (ne-PLND) with regard to oncologic outcomes. However, that support was mainly based on retrospective, non-randomized studies with potential bias [7, 8]. Recently, a prospective, randomized clinical trial (RCT) evaluated the survival benefits and the harm of super-extended PLND (se-PLND). The upper boundary of the se-PLND extended to the para-aortal nodes including the inferior mesenteric artery (IMA). The RCT was designed to assess whether se-PLND prolonged 15% recurrence-free survival (RFS) in comparison with standard PLND, but it failed to demonstrate a significant reduction of recurrence within the expected range [9]. Herein, this meta-analysis was conducted in order to assess the value of e-PLND in both short- and long-term outcomes for patients with bladder cancer undergoing curative surgeries.

## Materials and methods

### Searching strategies

This meta-analysis was conducted in accordance with the Preferred Reporting Items for Systematic Review and Meta-Analyses statement. We searched PubMed, EMBASE, and the Cochrane Library databases for relevant works in the English language from the database inception to June 10, 2019, using combinations of the following keywords: (“radical cystectomy” OR “bladder cancer” OR “urinary bladder carcinoma”) AND (“lymph node dissection” OR “lymphadenectomy”). References from retrieved articles were checked for any additional studies. We used data only from full-published articles. Meeting or conference abstracts were excluded.

### Study inclusion and exclusion criteria

**Fig. 1:**
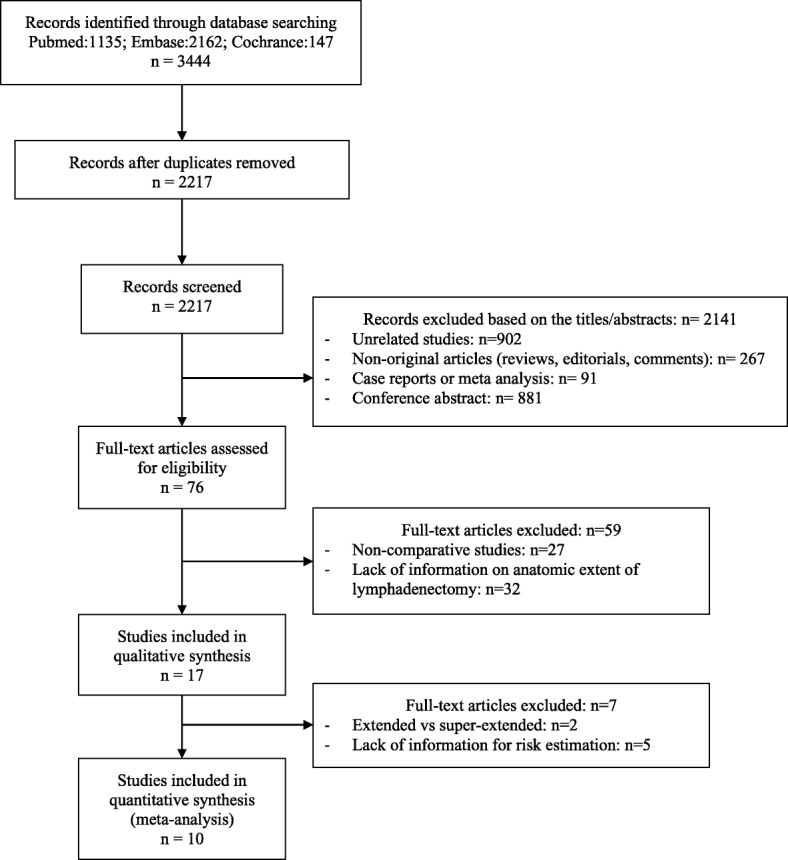
Flow diagram identifying eligible studies and exclusion criteria

Two reviewers (Yu-Chen Wang and Jie Wu) independently selected studies by an initial screen of identified abstracts or titles and a second screen of full-text articles. Studies were considered eligible if they met the following criteria: (1) the study was a RCT or cohort study, (2) explicit description of the boundaries of the LND performed during RC was provided, and (3) risk estimates with corresponding 95% CIs were reported, or sufficient data were provided to calculate corresponding values. We excluded studies that did not meet the inclusion criteria (Fig. 1).

### Outcomes of interest

The primary outcome was RFS. Secondary outcomes included disease-specific survival (DSS), overall survival (OS), rates of complications occurring within 90 days after the surgery, and mortality. In terms of complications, we used rates of postoperative complication classified by Clavien-Dindo grades. Rates of major complications (Clavien grade > 2) and mortality (Clavien grade = 5) were extracted and grouped into early events (0–30 days) and late events (31–90 days) based on postoperative period.

### Data extraction

Two authors independently extracted data from primary texts and supplementary appendices using data abstraction forms. The following information was extracted: author, year of publication, study design, follow-up time, explicit description of PLND template, sample size, median number of dissected nodes, use of adjuvant or neoadjuvant therapy, and other outcomes of interest. PLND template was classified as e-PLND or ne-PLND based on whether the proximal extent of PLND was higher than the level of the bifurcation of iliac vessels or not. For risk estimation of survival data, results from the main multivariable model were used that included the most adjusted confounders were used. Otherwise, estimations, based on univariate cox models, were used. When hazard ratios (HRs) were not directly documented in the literature, Engauge Digitizer V4.1 (Markmitch, Goteborg, Sweden) was used to digitize Kaplan-Meier curves. In combination with a previously described approach [10], we then estimated HRs and corresponding statistics. In addition, we also extracted estimated 5-year RFS rates for both the ne-PLND group and the e-PLND group.

### Quality assessment

According to the Oxford Centre for Evidence-Based Medicine criteria [11], two reviewers independently rated the level of evidence for all eligible studies. Herein, methodological quality of the studies was assessed based on the Newcastle-Ottawa scale for observational comparative studies [12].

### Statistical analysis

Data analyses were performed using RevMan software 5.3 (Cochrane Collaboration, Oxford, UK) and the “meta” package in R software 3.6.0 (R Foundation for Statistical Computing, Vienna, Austria) [13]. Odds ratios (ORs) were used to compare dichotomous variables. The natural logarithm of HRs and the corresponding standard error were used for the meta-analysis of survival data [14]. We tested heterogeneity by Cochran’s *Q* test and Higgins *I*^2^ statistic. A *P* value by *Q* test of less than 0.10 and *I*^2^ > 50% indicated existence of statistically significant heterogeneity. A random-effects model was used for outcomes when heterogeneity existed; otherwise, a fixed-effects model was used. Sensitivity analysis was conducted by sequentially omitting each individual study in order to evaluate the stability of the synthetic results. We also assessed publication bias using contour-enhanced funnel plot [15] with a *P* value lower than 0.05 for Egger’s test indicating significant statistical publication bias [16]. All results were reported with 95% confidence intervals (CIs), and a two-sided *P* value of < 0.05 was considered statistically significant.

## Results

The process of identifying eligible studies is shown by a flow chart (Fig. 1). A total of 3444 records were identified based on primary search strategies. After excluding unrelated studies, duplicate reports, narrative reviews, and conference abstracts, we reviewed the full text of 76 articles at length. We then identified 59 articles as ineligible in that the articles either were non-comparative or lacked an exact description of the anatomic extent of PLND. Among the remaining seventeen studies, 7 studies were excluded from the final meta-analysis because no comparison was made of extended PLND to super-extended PLND or there was a lack of quantitative information on outcomes of interest. A total of 10 studies including 3979 cases fulfilled the predefined inclusion criteria and were used in the final quantitative synthesis. The two reviewers reached complete agreement on study selection and 95% agreement on quality assessment of the studies.

### Characteristics of included studies

Of all eligible studies, one was prospective and a RCT [9]. One study was a prospective collection of patient data [17]. The remaining were retrospective [18–26]. There were 1924 patients who underwent extended PLND with the proximal extent of PLND ranging from the aortic bifurcation to the inferior mesenteric artery. Another 2497 patients underwent more limited PLND with cranial boundaries limited at the bifurcation of the common iliac arteries or lower. Basic characteristics of the 10 studies are summarized in Table 1.

**Table 1:** Basic characteristics of included studies and long-term survival in both extended and non-extended LND groups

Author	Year	Study design	Type of LND		Number of cases		Median number of removed LNs		Follow-up, month (C/I)	Neoadjuvant or adjuvant therapy	NOS grade	Evidence level
			C	I	C	I	C	I				
Poulsen	1998	Cohort studies	sLND	eLND	68	126	14	25	61.7/23.5	None	9	2b
Dhar	2008	Cohort studies	lLND	eLND	336	322	12	22	51/36	NR	8	2b
Abol-Enein	2011	Cohort studies	sLND	seLND	200	200	16	49	50.2	None	9	2b
Holmer	2009	Cohort studies	lLND	eLND	69	101	8	37	94/38	Adjuvant CT	9	2b
Hugen	2010	Cohort studies	sLND	seLND	206	54	9	46	NR	NR	7	2b
Jensen^a^	2012	Cohort studies	lLND	mixed	204	265	6	23	113/45	None	9	2b
Simone	2012	Cohort studies	sLND	eLND	584	349	18	29	96	Adjuvant CT/RT	9	2b
Gschwend	2018	RCT	sLND	seLND	203	198	19	31	43	Adjuvant CT	9	1b
Adbi	2016	Cohort studies	sLND	eLND	105	105	9	21	18/19	Neoadjuvant CT	8	2b
Andrea	2019	Cohort studies	sLND	Mixed	200	34	13	NR	NR	NR	9	2b

*C* control group, *I* intervention group, *LN* lymph node, *CT* chemotherapy, *RT* radiotherapy, *NR* not recorded, *lLND* = limited LND, *sLND* standard LND, *eLND* extended LND, *seLND* super-extended LND, *mixed* a mixture of extended and super-extended LND, *RCT* randomized controlled trial, *NOS* Newcastle-Ottawa scale

^a^The number of removed LNs was the mean number in each group

For postoperative complications after curative RC with PLND, three studies reported the rate of major complications within 90 days after surgery [9, 18, 27]. Rates of early postoperative mortality, defined as death by all causes within 30 days, were presented in three studies [9, 18, 27], while rates of mortality between 31 and 90 days were presented in two studies [9, 27].

### Long-term survival

**Fig. 2:**
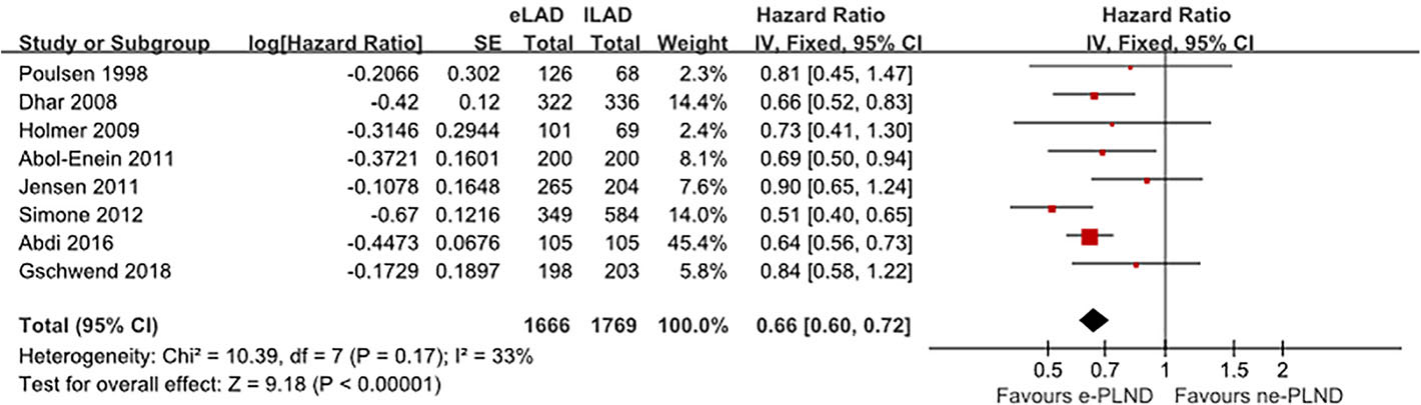
Meta-analysis of recurrence-free survival

**Fig. 3:**
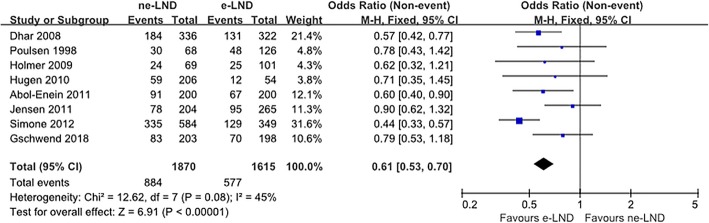
Meta-analysis of 5-year recurrence rate

We managed to extract results from nine studies that assessed a difference in RFS between the e-PLND group and the ne-PLND group. HR for RFS and 5-year RFS rates were available in eight and seven studies, respectively. The extended PLND template associated with a significantly better RFS (HR 0.74, 95% CI 0.62–0.90, *p* = 0.002, Fig. 2). Moreover, we found a significant correlation between the PLND template and higher 5-year RFS rate (OR 0.61, 95% CI 0.53–0.70, *p* < 0.001, Fig. 3).

**Fig. 4:**
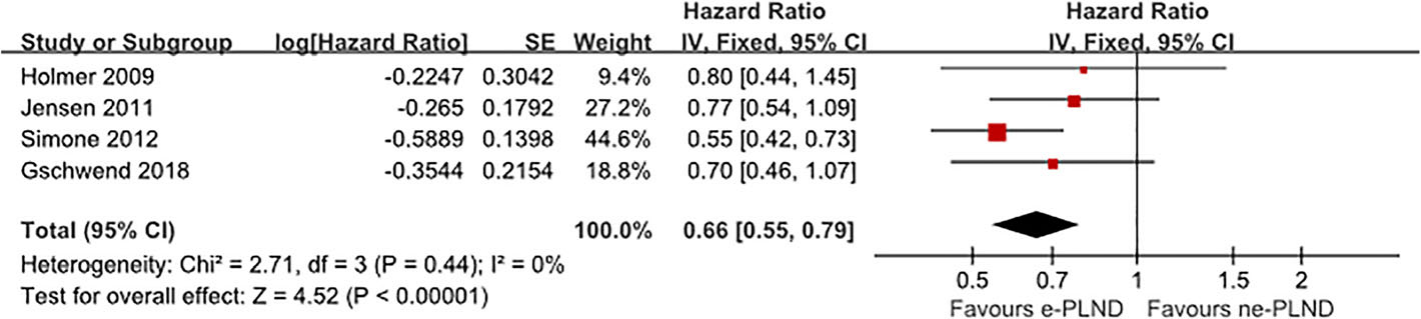
Meta-analysis of disease-specific survival

With respect to DSS, four studies involving 1973 patients evaluated the impact of the proximal extent of PLND. After collectively pooling the data from all studies, we found a significant correlation between e-PLND and better DSS (HR 0.66, 95% CI 0.55–0.79, *p* < 0.001, Fig. 4).

**Fig. 5:**
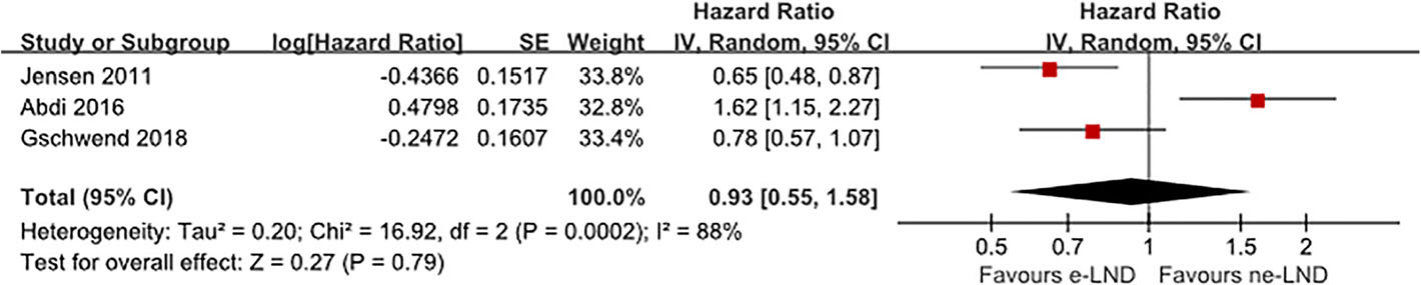
Meta-analysis of overall-survival

Four studies accessed the impact of LND template on OS with great heterogeneity (*I*^2^ = 84%, *p* < 0.001). The result showed no correlation between the e-PLND template and a better OS (HR 0.93, 95% CI 0.55–1.58, *p* = 0.79, Fig. 5).

### Major complications and short-term mortality

**Fig. 6:**
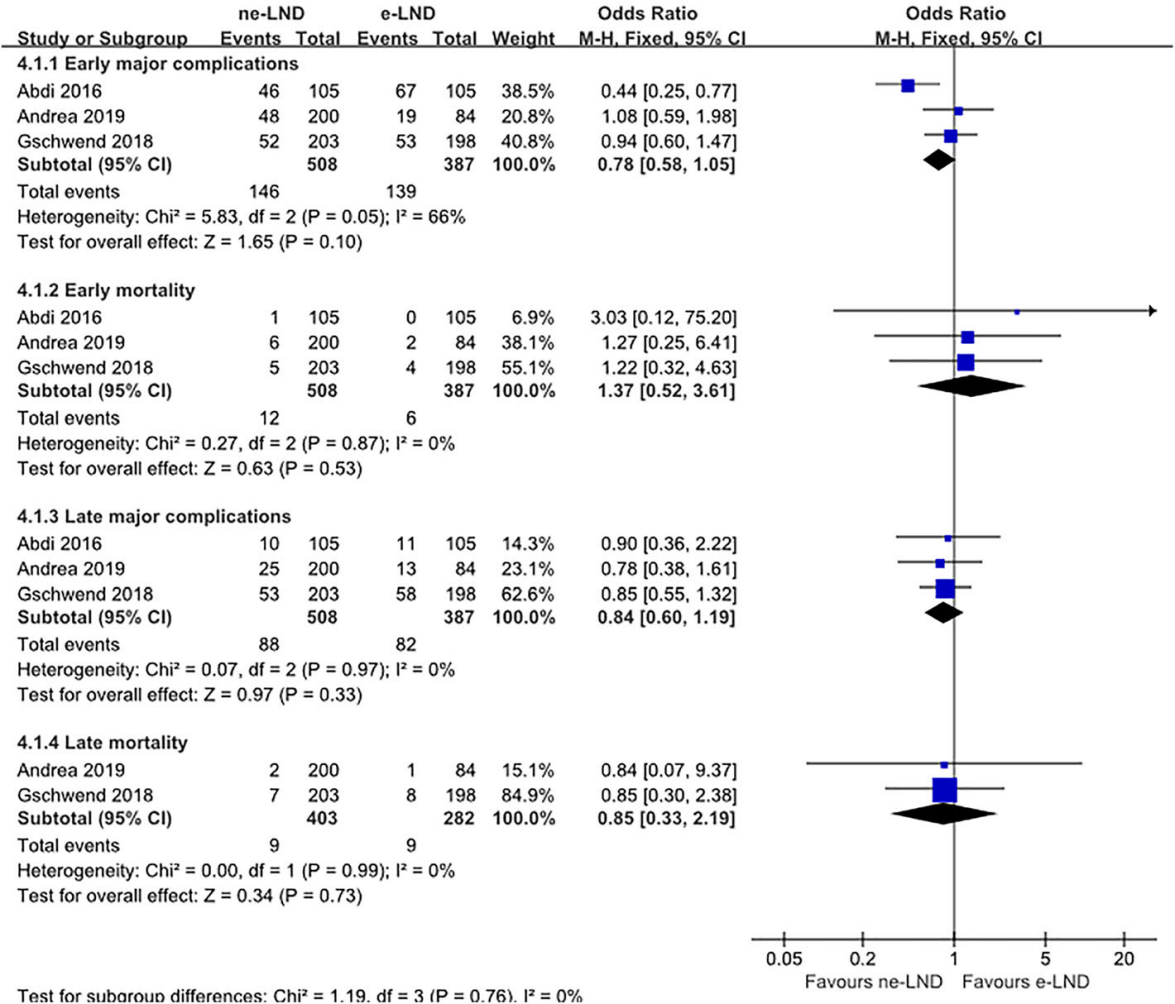
Meta-analysis of major complication rate and mortality within 90 days of surgery

Based on the Clavien-Dindo classification [28], three studies compared postoperative complication rates between patients who underwent extended PLND and those underwent non-extended PLND as well as compared mortality within 90 days after surgery [9, 18, 27]. Meta-analysis showed no significant difference in major complications nor short-term mortality between the e-LND group and the ne-LND group (Fig. 6).

### Sensitivity analysis and publication bias

**Fig. 7:**
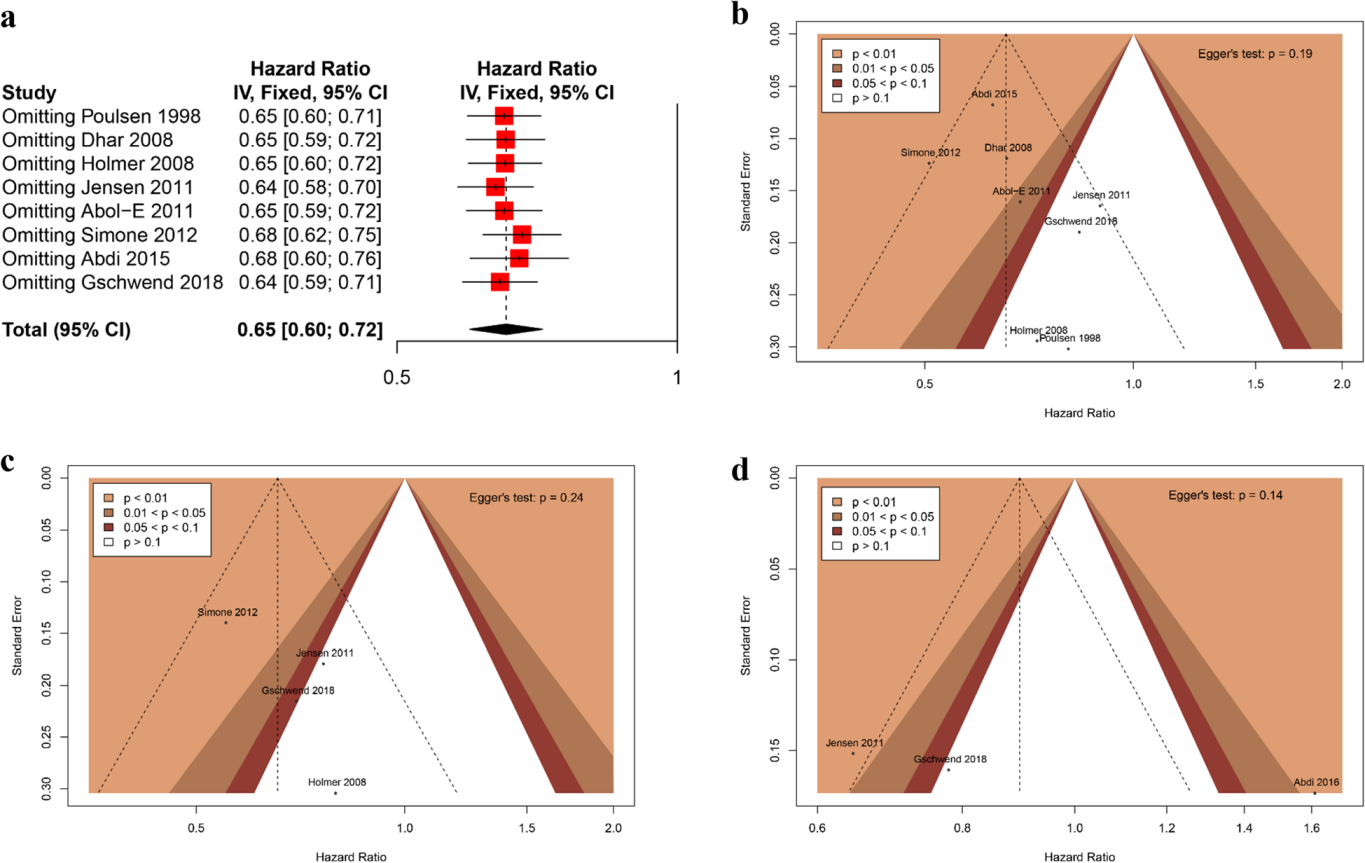
**a** The fixed-effects sensitivity analysis of recurrence-free survival. Contour-enhanced funnel plots and Egger’s test evaluating publication bias regarding recurrence-free survival (**b**), disease-specific survival (**c**), and overall-survival (**d**) in the meta-analysis

After sequentially removing each study in order to evaluate if any study had an exaggerated impact on the pooled HR for RFS, no obvious deviation was found and the results demonstrated that the synthetic evidence was robust (Fig. 7a). As shown in Fig. 7b, contour-enhanced funnel plots showed the absence of remarkable asymmetry and the *P* value of Egger’s test was 0.19. These results demonstrated no obvious publication bias.

## Discussion

The present meta-analysis was conducted to compare e-LND and LND of their impacts on postoperative complications and the survival benefits including RFS, DSS, and OS. The primary rationale for extended LND is elimination of micro-metastases residing within lymph nodes. Therefore, RFS was the primary outcome and results showed a significant improvement in RFS for patients undergoing e-LND, which is consistent with previous meta-analyses [7, 8]. A positive effect of e-LND on DSS was also observed. This was presumably due to the distribution of metastatic lymph nodes above the level of the iliac bifurcation, based on previous mapping studies [4–6]. Nearly 40% of LN-positive patients possess metastatic LNs above the upper boundary of regions described by standard LND templates [4]. These may be curable by meticulous LND. However, LND with a higher proximal extent was not superior with regard to OS. In these meta-analyses, three studies compared postoperative complication rates among different LND templates and found no significant differences in major complications or mortality within 90 days of surgery [9, 18, 27]. Therefore, a more extended LND procedure above the level of the iliac vessels may not be a risk factor to short-term survival. However, none of the studies specified the major complications in both groups. Further studies are needed to explore the differences in lethal complications between the extended and non-extended LND. Moreover, RCT with the primary endpoint being perioperative complications is required to confirm the safety of the extended LND template.

According to a large prospective study, conducted in two independent institutions, that assessed both the diagnostic and therapeutic values of extended LND, 41% of patients with node metastases had positive LNs above the common iliac bifurcation. This observation indicated the importance of an e-PLND. A comparative mapping study also demonstrated that both LN positivity and prognosis were similar between cohorts, despite a significant difference in median LN counts [4]. Results from the RCT demonstrated that more dissected lymph nodes were not associated with better outcomes [9]. Hence, LND template is of particular value for diagnosis and therapy.

With regard to the cephalad extent of LND, extended LND template and super-extended LND template were combined and named extended LND. This approach does not permit an answer to the question: which template leads to a greater survival benefit? Only a few studies assessed the impact of different extended LND templates on long-term survival. Møller retrospectively analyzed two cohorts of patients undergoing super-extended LND and extended LND, at the time of RC [29]. No significant differences were observed for RFS or DFS while a difference was observed for OS. However, patients in the se-PLND group were significantly older than the e-PLND group, which may have contributed to better OS for the latter group because age has been identified as a strong determinant of prognosis. The observation that an extended template up to the level of IMA failed to prolong RFS and DFS seemed counterintuitive for the overall population, though a tendency toward a better survival in the subset of patients with non-organ confined and node-negative disease was observed. Similar conclusions were reached by a retrospective study that was completed at two separate centers, where extended LND up to IMA and LND to the bifurcation of the aorta were performed respectively [2]. Further studies are required to clarify the optimal cranial extent of an extended LND template with regard to long-term survival. Based on the existing evidence identified by this meta-analysis, the nomenclature and criteria for the classification of an extended LND are reasonable and unlikely to cause significant bias.

In the setting of preoperative chemotherapy for patients clinically diagnosed with node-positive bladder cancer, Philip explored the variables that correlated with cancer-specific survival after consolidative surgery following upfront chemotherapy [19]. In this study, a more proximal extent of LND ranging from the bifurcation of the aorta to the renal helium was not associated with better survival when compared to a more limited LND confined to regions below the bifurcation of the common iliac vessels. On the contrary, retroperitoneal LND significantly correlated with poorer cancer-specific survival, possibly due to lymph node metastases involving the retroperitoneum at the time of diagnosis for the RPLND group. This negative correlation and the exclusion of patients with radiologic evidence of enlarged lymph nodes above the aortic bifurcation prior to suggests that more extended LND is not superior to limited LND with regard to long-term survival in patients with M1a nodal disease. Further, with the increasing importance of neoadjuvant chemotherapy in contemporary clinical treatment of MIBC, the exclusion of patients receiving pre-operative chemotherapy in most studies may obscure the true benefit of an extended LND template.

Currently, there is only one prospective RCT designed to compare the effect of standard versus super-extended LND during curative RC [9]. Despite the negative results attributed to several factors including the inclusion of patients with NMIBC, large numbers of dissected lymph nodes, and an imbalance in patients with T4 disease and positive lymph nodes, a trend for longer survival suggests that extended LND provides extra therapeutic benefit and facilitates RC. This meta-analysis found no remarkable improvement in overall survival, yet notable heterogeneity existed among the three studies. Another RCT dealing with similar issues was initiated by the Southwest Oncology Group. The results of that study should provide convincing evidence that will support clinician’s decision-making.

Despite several promising results, the findings of this study should be interpreted within the context of study limitations. First, most of the included studies were retrospective in design, which may bias the pooled estimates in favor of an extended PLND template. However, tests for publication bias revealed none and it was unlikely that more positive results have been published. Second, a relatively high level of heterogeneity was found when pooling estimates for OS. In most retrospective studies, the selection of the PLND template was left to the discretion of surgeons. This may lead to a higher proportion of patients with lymph node metastases above the level of the iliac vessels in the e-PLND group. Juxtaposed with earlier reports that patients with nodal disease at such a high level may not derive survival benefit from an extended LND [19, 30], the results may be biased for pooled HR for OS. Third, we used Tierney’s method to estimate HRs and 95% CI, which may have introduced bias.

## Conclusion

The results of this meta-analysis substantiate the favorable prognostic value of extended PLND for long-term survival of patients with bladder cancer. Similarly, short-term survival and postoperative complication rates indicate the safety of an extended PLND template.

## Data Availability

All the data used in the current study were collected from the corresponding published papers.

## Abbreviations

CI: Confidence interval
DSS: Disease-specific survival
e-PLND: Extended PLND
HR: Hazard ratio
IMA: Inferior mesenteric artery
LND: Lymph node dissection
MIBC: Muscle-invasive bladder cancer
ne-PLND: Non-extended PLND
OR: Odds ratio
OS: Overall survival
PLND: Pelvic lymphadenectomy
RC: Radical cystectomy
RCT: Randomized clinical trial
RFS: Recurrence-free survival
se-PLND: Super-extended PLND
